# Misleading Claim by Ben-Ami *et al.* (2014)

**DOI:** 10.1017/awf.2025.7

**Published:** 2025-03-11

**Authors:** Neal Andrew Finch, Peter Murray

**Affiliations:** University of Southern Queensland, School of Agriculture and Environmental Science, Toowoomba, QLD, Australia

It has recently come to our attention that an article published in *Animal Welfare* in 2014 makes a very poorly substantiated but significant claim that is being regularly cited in the scientific and grey literature.

Specifically, the statistic that 40% of kangaroos commercially harvested in Australia are not shot in the head is cited in an *Animal Welfare* article. We first heard this statistic used by Senator Tammy Franks in June 2024 in an address to the South Australian parliament. Senator Franks asked the parliament “*Would any other industry accept a wounding rate this high?*” As wildlife biologists who teach wildlife management, including the sustainable use of wildlife, we and a number of our colleagues found this statistic alarming and wanted to know the source. A quick search brought up many more examples of this statistic being quoted, in the last couple of years.

An example of the kind of citations where this statistic is used can be found here: https://www.eurogroupforanimals.org/what-we-do/areas-of-concern/imports-kangaroo-derived-products

The source of this statistic is the 2014 publication:

D Ben-Ami, K Boom, L Boronyak, C Townend, D Ramp, D Croft and M Bekoff 2014 The welfare ethics of the commercial killing of free-ranging kangaroos: an evaluation of the benefits and costs of the industry. *Animal Welfare* **23**: 1-10.

In this article the authors refer to two studies that have attempted to quantify how many kangaroos commercially harvested in Australia are not shot according to the National Code of Practice for the humane shooting of kangaroos and wallabies for commercial purposes (in the brain). The first study is highly credible and was conducted in 2002 by RSPCA Australia. In this study, 2,689 skins and 2,394 kangaroo carcases, from multiple sites across Australia, were inspected for the location of bullet holes. The authors concluded that approximately 4% of animals had not been shot in the head. Ben-Ami *et al.* (2014) then refer to another ‘study’ by Animal Liberation NSW giving it equal merit to the RSPCA research where up to 40% of kangaroos were reportedly not shot in the head. This ‘study’ is referenced in Ben-Ami (2009), a report commissioned by Animal Liberation NSW. This report is openly biased and not peer-reviewed, however by referencing his own work in Ben-Ami *et al.* (2014) this proposition, specifically that 40% of harvested kangaroos are not shot in the head, has been given peer-reviewed credibility. The source of this statistic in Ben-Ami (2009) is a self-proclaimed expert witness who allegedly examined 420 photographs taken of kangaroo carcases in field chillers in several locations. The witness claims to identify where the head of the kangaroo is removed based on 204 photographs. Of these, it is claimed 82 kangaroos, based on photographs, were not cut through the atlantal-occipital joint which is then assumed to mean the animal was not shot in the head.

Ben-Ami *et al.* (2014) state that the “*apparently large difference*” in the non-head-shot estimates is due to differences in sampling methodology. They make no mention that the RSPCA work was credible and validated whereas the Animal Liberation report is based on evidence gathered by people with the specific intent of discrediting an industry. To present such findings with equal merit in a peer-reviewed journal is deliberately misleading.

This would be a minor issue and possibly not worthy of bringing to your attention except for the fact that the statistic of 40% of kangaroos not shot in the head is currently being deliberately used to discredit the kangaroo industry. By referencing a peer-reviewed and credible journal, such as *Animal Welfare*, this unsubstantiated and questionable statistic is given unjustified validity.

The wounding rates of any harvesting regime is a serious issue. The RSPCA research published in 2002 was intended to assess any improvements by the industry since the earlier research of this nature published in 1985. The RSPCA reported a significant improvement by the industry whilst noting more improvements could be made. That research is now over two decades old, and we suggest new research should be conducted to establish if improvements have continued from the RSPCA conclusion that approximately 4% of animals had not been shot in the head. Until then, to use the statistic of 40% non-head shot kangaroos by quoting Ben-Ami *et al.* (2014) as a credible source is deliberately misleading.
